# Impaired ECM Remodeling and Macrophage Activity Define Necrosis and Regeneration Following Damage in Aged Skeletal Muscle

**DOI:** 10.3390/ijms21134575

**Published:** 2020-06-27

**Authors:** Fasih Ahmad Rahman, Sarah Anne Angus, Kyle Stokes, Phillip Karpowicz, Matthew Paul Krause

**Affiliations:** 1 Department of Kinesiology, University of Windsor. Windsor, ON N9B 3P4, Canada; fasih.rahman@uwaterloo.ca (F.A.R.); anguss@uwindsor.ca (S.A.A.); 2 Department of Biomedical Sciences, University of Windsor. Windsor, ON N9B 3P4, Canada; stokesk@uwindsor.ca (K.S.); Phillip.Karpowicz@uwindsor.ca (P.K.)

**Keywords:** skeletal muscle, regeneration, extracellular matrix, macrophage, plasminogen activator inhibitor-1, aging

## Abstract

Regenerative capacity of skeletal muscle declines with age, the cause of which remains largely unknown. We investigated extracellular matrix (ECM) proteins and their regulators during early regeneration timepoints to define a link between aberrant ECM remodeling, and impaired aged muscle regeneration. The regeneration process was compared in young (three month old) and aged (18 month old) C56BL/6J mice at 3, 5, and 7 days following cardiotoxin-induced damage to the tibialis anterior muscle. Immunohistochemical analyses were performed to assess regenerative capacity, ECM remodeling, and the macrophage response in relation to plasminogen activator inhibitor-1 (PAI-1), matrix metalloproteinase-9 (MMP-9), and ECM protein expression. The regeneration process was impaired in aged muscle. Greater intracellular and extramyocellular PAI-1 expression was found in aged muscle. Collagen I was found to accumulate in necrotic regions, while macrophage infiltration was delayed in regenerating regions of aged muscle. Young muscle expressed higher levels of MMP-9 early in the regeneration process that primarily colocalized with macrophages, but this expression was reduced in aged muscle. Our results indicate that ECM remodeling is impaired at early time points following muscle damage, likely a result of elevated expression of the major inhibitor of ECM breakdown, PAI-1, and consequent suppression of the macrophage, MMP-9, and myogenic responses.

## 1. Introduction

Skeletal muscle constitutes approximately 40% of the total mass of the human body and plays a central role in health and well-being [1]. With aging, a combination of neurodegeneration, an altered hormonal and metabolic profile, and reduced regenerative capacity leads to a deterioration of skeletal muscle culminating in loss of muscle mass, quality, and function. Although diet, exercise, and pharmacological treatments show promise in limiting such detrimental developments [2,3,4,5,6], our understanding of the network of mechanisms involved in age-related muscle wasting remains limited. Central to the maintenance of a healthy skeletal muscle mass is its regenerative capacity, enabling muscle to completely restore function within 7–10 days after severe damage [7]. The regeneration process can be categorized into the following three sequential but widely overlapping stages: (1) inflammation and necrosis of damaged myofibres, (2) activation, proliferation, differentiation, and fusion of satellite cells, and (3) maturation and remodeling of the regenerated muscle [7,8,9,10]. Each stage is essential to drive the following subsequent stage, thereby imparting coherence to the overall regeneration process.

The extracellular matrix (ECM) is critical in maintaining normal skeletal muscle function and driving skeletal muscle regeneration. Skeletal muscle ECM is composed of a plethora of structural, adhesion, and signal-stimulating proteins that are transiently degraded and reconstituted depending on the mode and severity of tissue injury [9,10]. Aged skeletal muscle does not regenerate well in response to injury, and there is evidence of impairment at each stage of the regeneration process [10,11,12] including accumulation of collagen (i.e., fibrosis) [13,14,15,16,17,18]. However, it is unclear if this age-related skeletal muscle fibrosis occurs as a result of impaired degradation in the first week following tissue damage.

**Figure 1:**
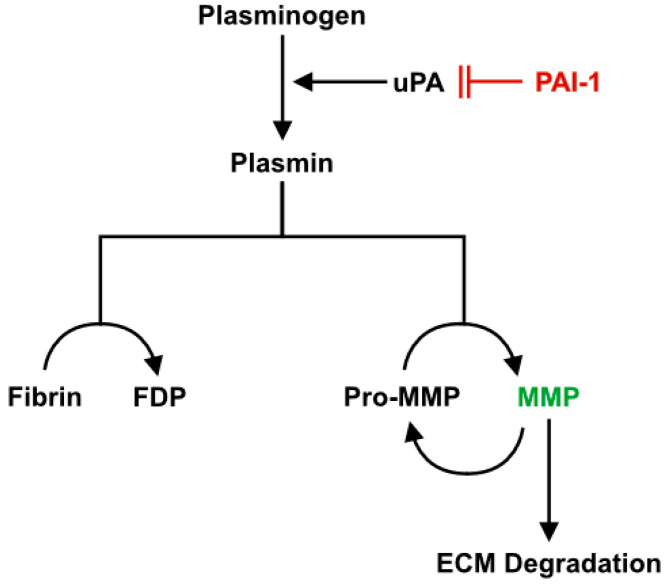
Plasminogen system. Plasminogen is the inactive substrate of the plasminogen system that is activated into plasmin primarily by urokinase plasminogen activator (uPA) in skeletal muscle. Plasmin can directly degrade fibrin into fibrin degradation products (FDPs) and activate MMPs. The MMPs work to degrade connective tissue in the ECM and also activate additional MMPs in a positive feedback loop. PAI-1 functions as the upstream inhibitor of the plasminogen system by inhibiting uPA and thus preventing the activation of plasminogen. Adapted from Higazi, A.A.-R [32].

Plasminogen Activator Inhibitor-1 (PAI-1), a widely circulated protein (approximately 15–550 ng/mL in the blood [19]) that is responsible for the inhibition of the plasminogen system (Figure 1) [20,21], is found in greater concentration in the circulation with advancing age [22,23,24]. The plasminogen system is responsible for the remodeling of the ECM through the degradation of fibrous proteins [25,26,27,28] and thus plays a critical role during skeletal muscle regeneration. An active plasminogen system turns on several proteolytically active enzymes, including plasmin, which then activates certain matrix metalloproteinases (MMPs) by cleaving off an inhibitory peptide. Activated MMPs are responsible for the direct degradation of proteins within the ECM, particularly during massive remodeling events such as tissue regeneration. During skeletal muscle regeneration, PAI-1 has been demonstrated to impair infiltration of macrophages, inhibit activity of MMP-9, while allowing accumulation of ECM, thus limiting the rate of regeneration [29,30,31]; however, the role of PAI-1 in age-related regeneration impairments has yet to be investigated. Given that PAI-1 can impede muscle regeneration and ECM remodeling, it is of interest to know if age-related detriments to skeletal muscle regeneration and increased ECM deposition are related to elevations in PAI-1. Furthermore, it is unknown if the effects of PAI-1 on muscle regeneration are related to systemic or local expression of PAI-1. Thus, we hypothesized that aged skeletal muscle would express greater PAI-1 in mouse skeletal muscle during regeneration and demonstrate poor ECM remodeling, macrophage infiltration, and secretion of MMP-9. We utilized an acute time course of 3, 5, and 7 days following muscle damage to histologically examine the effects of age on skeletal muscle regeneration. We found that aged muscle exhibits elevated expression of PAI-1, poor ECM remodeling, and aberrant macrophage-related expression of MMP-9 following induction of muscle damage. Furthermore, aged muscle was unable to adequately regenerate in comparison with young muscle.

## 2. Results

### 2.1. Body Mass, Muscle Mass, and Myofiber Cross-Sectional Area

**Figure 2:**
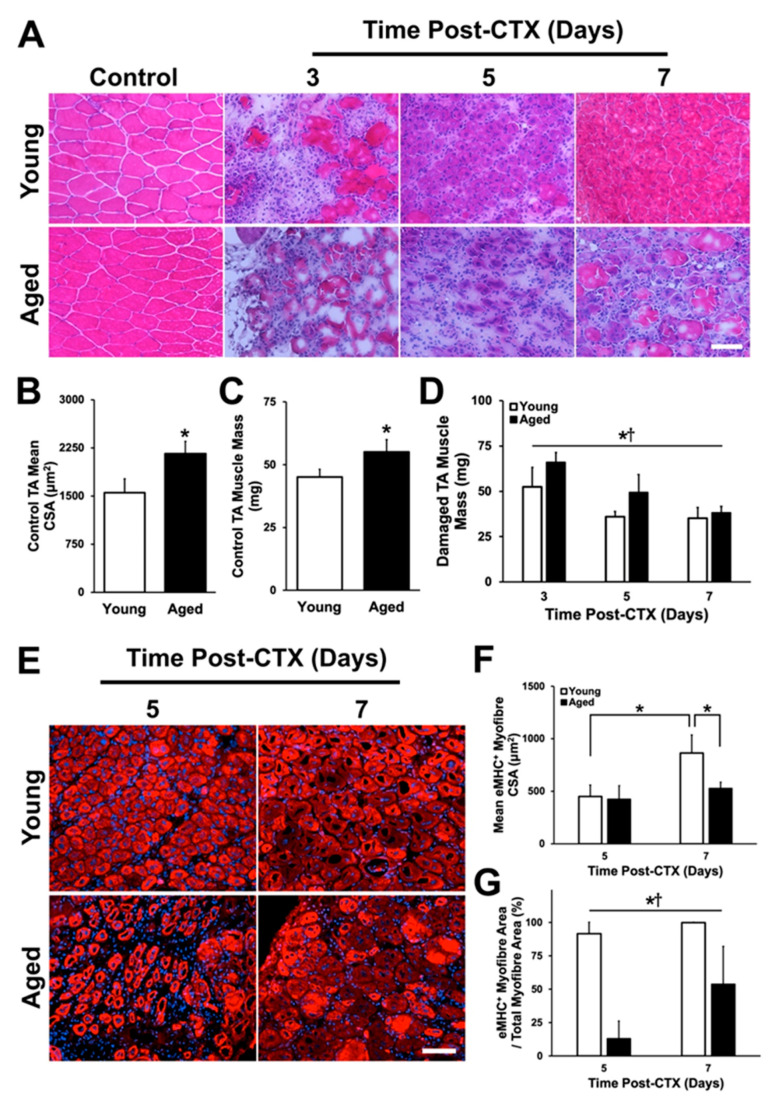
Regenerative capacity of aged skeletal muscle is impaired. (**A**) hematoxylin and eosin (H&E) cryosections of the TA muscle throughout the regeneration time course (3–7 days). Control (undamaged) TA (**B**) mean cross-sectional area (*p* = 0.003) and (**C**) muscle mass were significantly greater in the aged group (*p* < 0.001). * denotes a significant difference detected by two-tailed *t*-test. (**D**) Damaged TA muscle mass was different between young and aged groups and across recovery time points. * denotes a significant main effect of age (*p* = 0.001). † denotes a significant main effect of recovery time point following damage (*p* < 0.001). (**E**) Regenerating myofibers were identified via embryonic myosin heavy chain (eMHC; red) and 4,6-diamidino-2-phenylindole (DAPI; blue) at five and seven days following damage. (**F**) A significant interaction between age and recovery time points was observed (*p* = 0.02). Regenerating myofiber cross-sectional area was found to increase from five to seven days in young mice (simple main effects post-hoc analysis: *p* < 0.001), and there was a significant difference at seven days between young and aged muscle (simple main effects post-hoc analysis: *p* = 0.001). (**G**) Relative regenerating myofiber area was significantly greater in young muscle compared to aged muscle at five and seven days following damage. * denotes a significant main effect of age (*p* < 0.001). † denotes a significant main effect of recovery time point following damage (*p* = 0.013). *n* = 4–5 per group. Data presented are means ± standard deviation. Scale bar represents 50 μm.

To investigate the acute regenerative capacity of aged skeletal muscle, we first induced damaged to the left tibialis anterior (TA) muscle via cardiotoxin (CTX) injection and analyzed samples after 3, 5, and 7 days. The right TA muscle remained undamaged and was used as a control as needed. Body mass of mice was determined at the time of sacrifice. Young mice weighed 25.1 ± 1.7 g while aged mice weighed 32.0 ± 2.5 g (*t*-test: *p* < 0.001). Assessment of the undamaged leg (Figure 2A) revealed a significantly larger mean cross-sectional area of the undamaged TA myofibers in aged mice (*t*-test: *p* = 0.003; Figure 2B). Undamaged TA muscle mass was found to be larger in aged mice as well (*t*-test: *p* < 0.001; Figure 2C). Damaged TA muscle mass differed between young and aged mice (main effect of age: *p* = 0.001; Figure 2D) but decreased in both groups over time across the recovery time (main effect of time: *p* < 0.001; Figure 2D). Muscle mass expressed relative to body mass was not different between young and aged (*p* > 0.05) but decreased over time (Figure S1; main effect of time: *p* < 0.001).

### 2.2. Assessment of Regenerative Capacity

Regeneration of damaged skeletal muscle involves the de novo formation of myofibers from the fusion of myoblasts. Regenerating myofibers express embryonic myosin heavy chain (eMHC) and have centrally located nuclei, making it possible to identify them for analysis (Figure 2E). No regenerating myofibers were present at three days following damage in young or aged muscle, thus, only five- and seven-day time points were analyzed. A significant interaction was found between groups and across the five- and seven-day time points (*p* = 0.02). No differences in mean regenerating myofiber cross-sectional area between groups was found at five days following damage; however, young regenerating myofiber cross-sectional area increased significantly between five and seven days following damage (post-hoc: *p* < 0.001; Figure 2F). A similar increase in cross-sectional area was not observed in aged muscle, and at seven days following damage, the cross-sectional area of young regenerating myofiber exceeded that of the aged regenerating myofiber (post-hoc: *p* = 0.001). The proportion of area occupied by regenerating myofibers relative to total damaged area was assessed to determine the regenerative response of the muscles following damage (Figure 2G). Here, total damaged area was defined as the summated area occupied by eMHC+ regenerating myofibers plus the summated area occupied by necrotic myofibers. The results from this analysis showed a significant main effect of age (*p* < 0.001) and recovery time (*p* = 0.013). Nearly all the damaged area was regenerating in young muscle at five and seven days following damaged (92% and 99%, respectively), compared to aged muscle (13% and 54%, respectively). Although there was an increase between five and seven days following damage in aged muscle (main effect of recovery time: *p* = 0.013), it still severely lacked behind that of young muscle. Centrally located nuclei per regenerating myofiber was also determined. Young regenerating mouse TA had more centrally located nuclei per myofiber (five days: 1.34 ± 0.12; seven days: 1.50 ± 0.11) compared to aged TA (five days: 1.07 ± 0.08; seven days: 1.17 ± 0.05; significant main effect of age: *p* < 0.001).

**Figure 3:**
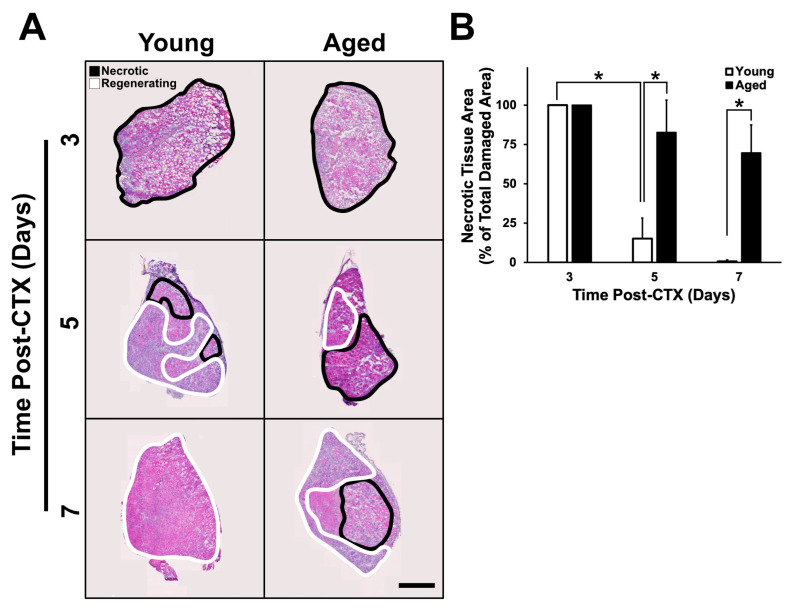
Necrosis is increased in aged muscle regeneration. (**A**) Representative images of whole muscle sections undergoing regeneration at their respective time points. (**B**) A significant interaction between age and recovery time points was identified (*p* < 0.001). Necrotic regions were found to be identical three days following damage in young and aged muscle, however, necrotic area decreased significantly in young muscle at five days following damage (simple main effects post-hoc analysis: *p* < 0.001 in all instances). The * indicates significant differences detected by a simple main effects post-hoc analysis (*p* < 0.001). *n* = 4–5 per group. Data presented are means ± standard deviation. Scale bar represents 500μm.

Hematoxylin and eosin (H&E)-stained muscle cross-sections were assessed to determine how quickly necrotic tissue was replaced with regenerating tissue. To do this, the proportion of necrotic area relative to total damaged area was determined. Necrotic regions are characterized by the presence of degenerating myofibers and an absence of newly regenerated myofibers containing centrally located nuclei [29,33,34]. Regions that eluded CTX-induced damage, identified by the presence of continuous neighboring undamaged myofibers, were not included in the analysis. A significant interaction was detected between groups and through the recovery time on presence of necrotic area within the tissue (*p* < 0.001). At three days following CTX treatment, damaged regions were entirely necrotic in both young and aged muscle (Figure 3A,B). Between three and five days following damage, young muscle exhibited a significant decline in necrotic area (post-hoc: *p* < 0.001); however, the same was not observed in the aged muscle. Additionally, at five and seven days following damage, young muscle was found to have significantly less necrotic tissue compared to aged muscle (post-hoc: *p* < 0.001). After observing these broad differences in necrotic and regenerating tissue in young and aged muscle, all subsequent analyses were characterized with respect to these distinct regions. 

### 2.3. Assessment of ECM Remodeling

Previous studies that have examined the accumulation of structural extracellular protein content in skeletal muscle have made limited conclusions regarding which specific components of the ECM exhibit changes with age [14,35,36,37,38]. Thus, an objective of this study was to determine whether changes in the ECM is consistent across different ECM proteins. This is a relevant question since different isoforms of collagen and other ECM components are expressed by different cell types and serve different roles in skeletal muscle. Collagens I and IV were selected for study because of their well-characterized roles in skeletal muscle. Collagen I is the most abundant form of collagen within the body and is distributed across the entire muscle tissue, providing structural support to the myofibers and the entire muscle group [39]. Collagen IV is specific to the basement membrane surrounding individual myofibers and provides structural support for individual myofibers while also forming a critical component of the satellite cell niche [40,41,42]. To assess the effect of age on collagens I and IV levels following muscle damage, the proportion of area exhibiting positive staining for each collagen isoform was quantified in both the necrotic and regenerating regions.

**Figure 4:**
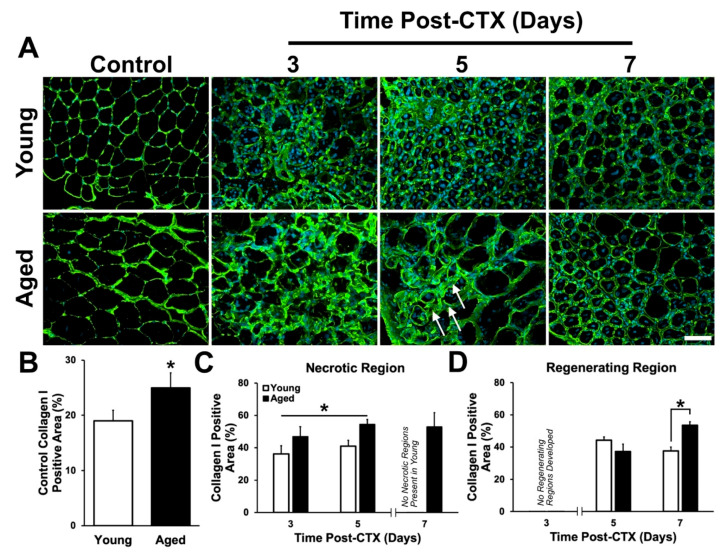
Collagen I expression is greater in the necrotic regions of aged muscle. (**A**) Immunostaining of collagen I (green) and DAPI (blue) at each time point following damage. Arrows denote regions of dense collagen I persistent at 5 days post-CTX in aged muscle. (**B**) Collagen I positive area in the undamaged contralateral leg was observed to be greater in aged muscle compared to young (*t*-test: *p* = 0.006; indicated by *). (**C**) Percent positive area of collagen I was greater in the necrotic region of aged muscle at three and five days following damage. * denotes a significant main effect of age (*p* = 0.002). (**D**) A significant interaction between age and recovery time points was observed (*p* = 0.034). Simple main effect post-hoc analysis demonstrates a significantly greater accumulation of collagen I in the regenerating region of aged muscle at seven days following damage (*p* = 0.024). * indicates significant differences detected by a simple main effects post-hoc analysis (*p* = 0.024). *n* = 4–5 per group. Data presented are means ± standard deviation. Scale bar represents 50 μm.

In undamaged TA muscles, collagen I content (Figure 4A) was found to be greater in the aged group compared to young (*t*-test: *p* = 0.006; Figure 4B). Following CTX-induced muscle damage, collagen I content increased in both young and aged muscle; however, collagen content within aged muscle remained significantly greater in the necrotic regions (main effect of age: *p* = 0.002; Figure 4C). A significant interaction was observed between age and recovery time points (*p* = 0.034; Figure 4D). A greater accumulation of collagen I was found at seven days following damage (post-hoc: *p* = 0.024; Figure 4D). The higher collagen I content in undamaged, necrotic, and late regenerating muscle acutely following damage is consistent with previous studies examining histochemically stained muscle tissue [35,37].

**Figure 5:**
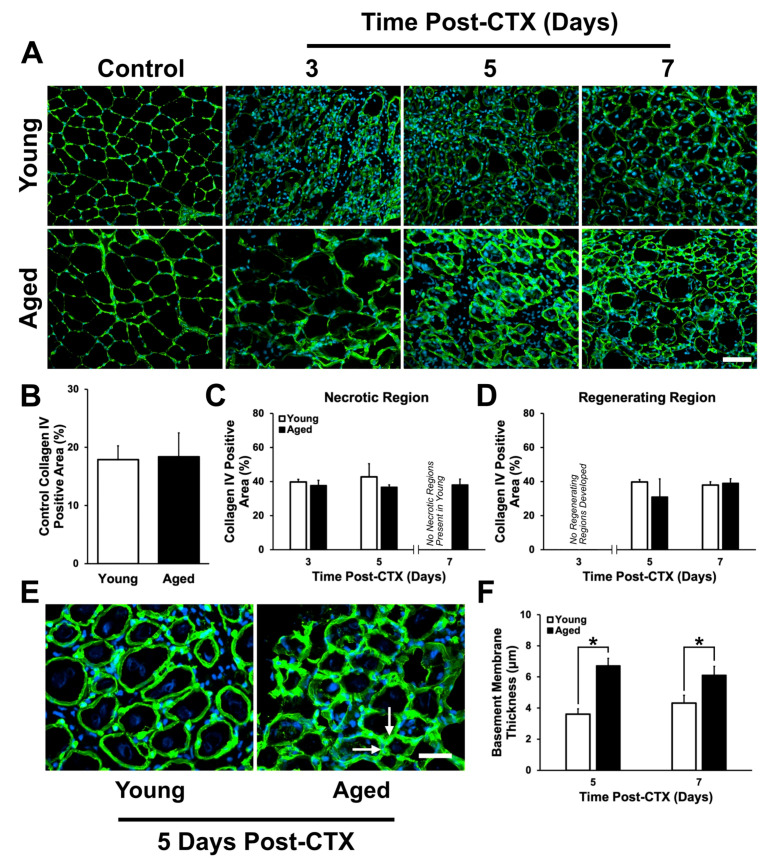
Collagen IV expression following cardiotoxin (CTX)-induced muscle damage in young and aged skeletal muscle. (**A**) Immunostaining of collagen IV (green) and DAPI (blue) at each time point following damage. (**B**) Collagen IV positive area in the undamaged contralateral leg was not significantly difference in young and aged muscle (*t*-test: *p* = 0.83). No significant differences in collagen IV percent positive area were detected in (**C**) necrotic and (**D**) regenerating regions. (**E**) Magnified of collagen IV area in young and aged muscle. Arrows denote regions of thicker collagen IV layers at five days post-CTX in aged muscle. (**F**) A significant interaction between age and recovery time point was observed (*p* = 0.015). Simple main effects post-hoc analysis found aged muscle to have a significantly greater collagen IV layer thickness compared to young at five and seven days following damage (*p* < 0.001 in both instances). * indicates significant differences detected by a simple main effects post-hoc analysis (*p* < 0.001). *n* = 4–5 per group. Data presented are means ± standard deviation. Scale bar represents 50 μm.

Collagen IV was investigated due to its role as a principle component of the basal lamina in the basement membrane. Area analysis of collagen IV (Figure 5A) revealed no difference in control or damaged TA muscles (Figure 5B–D) between young and aged mice. Although no differences in the proportion of collagen IV-positive area were found, it was noted that collagen IV structures appeared thicker and more irregularly shaped in regenerating aged muscle (Figure 5E). To analyze basal lamina thickness, collagen IV width was measured at five equally spaced intervals surrounding individual regenerating myofibers. A significant interaction was observed (*p* = 0.015) and a simple main effects post-hoc analysis demonstrated that significantly thicker collagen IV bands were found around aged regenerating myofibers compared to young (*p* < 0.001 between young and aged at five and seven days following damage; Figure 5F).

**Figure 6:**
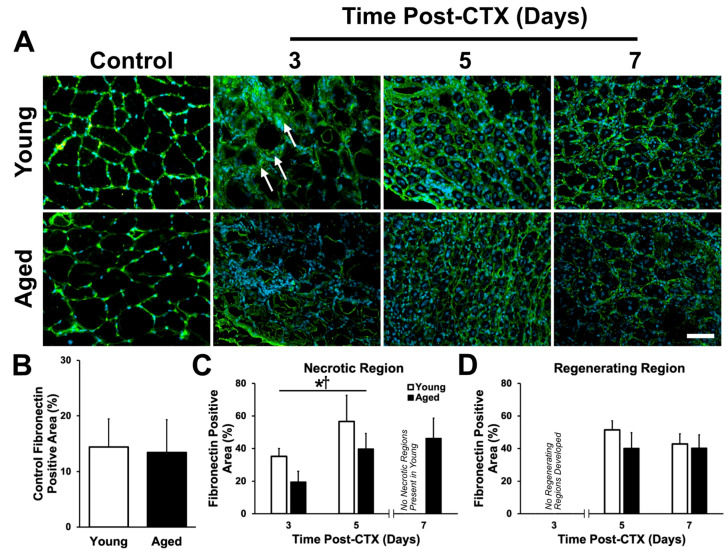
Fibronectin content is acutely greater in the necrotic region of young muscle. (**A**) Immunostaining of fibronectin (green) and DAPI (blue) at each time point following damage. Arrows denote regions of broad fibronectin at three days post-CTX in young muscle. (**B**) Fibronectin positive area in the undamaged control TA was not significantly different between young and aged (*t*-test: *p* = 0.256). (**C**) Fibronectin content increases over time from three to five days following damage in the necrotic region. Additionally, fibronectin was greater in the young groups during these acute time points. * denotes a significant main effect of age (*p* = 0.002). † denotes a significant main effect of recovery time point following damage (*p* < 0.001). (**D**) No statistically significant changes were observed in fibronectin content in the regenerating region of young and aged muscle. Note that the seven-day time point in the necrotic region and the three-day time point in the regenerating regions were not used in the statistical analysis due to insufficient instances of those regions depending on age and time point. *n* = 4–5 per group. Data presented are means ± standard deviation. Scale bar represents 50μm.

Fibronectin was also investigated. This protein serves as a link between collagen IV and integrins embedded in the myofiber membrane. Given the delay in regeneration characterized by low eMHC-expressing myofibers (Figure 2) and the well-defined role fibronectin plays in satellite cell proliferation, migration, and fusion [43,44], we hypothesized that aberrant fibronectin expression would contribute to impaired regeneration in aged muscle. Analysis of fibronectin immunostains (Figure 6A) demonstrated no differences between groups in undamaged TA muscles (*t*-test: *p* = 0.256; Figure 6B). In the damaged TA muscles, fibronectin expression was found to be greater in the necrotic regions of young muscle during all time points (main effect of age: *p* = 0.002; Figure 6C). Additionally, the expression of fibronectin increased between three and five days following damage in the necrotic regions of both aged and young muscle (main effect of time: *p* < 0.001; Figure 6C). No changes in fibronectin expression were observed in the regenerating regions between groups (*p* > 0.05; Figure 6D).

### 2.4. Macrophage Density Following Damage

**Figure 7:**
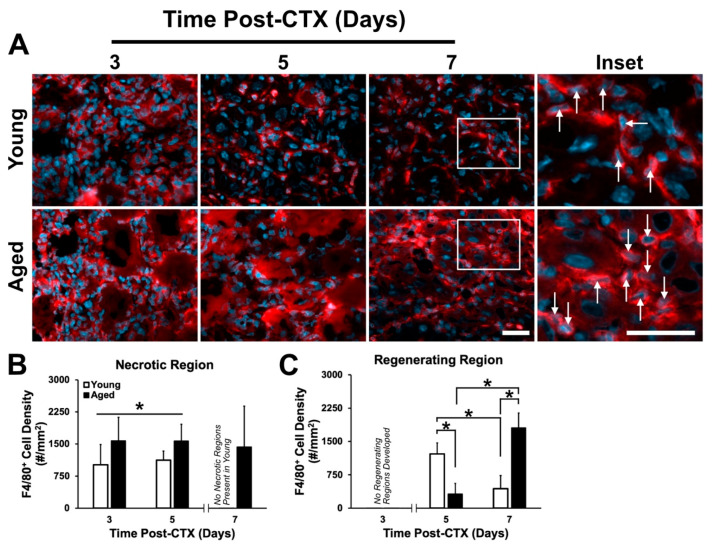
Macrophage responses to skeletal muscle differ between young and aged skeletal muscle. (**A**) Immunostaining of F4/80 (red) and DAPI (blue) used to identify macrophages. White arrows indicate F4/80+ cells (macrophages). White frame indicates region of higher magnification shown in neighbouring inset. Scale bars both represent 50 μm. (**B**) Macrophage density was greater in the necrotic region of aged muscle and remains steady between three to five days following damage. * denotes significant main effect of age (*p* = 0.025). (**C**) A significant interaction between age and recovery time point following damage was observed (*p* < 0.001). Simple main effect post-hoc analysis revealed a significant difference in macrophage density at five days following damage, with young muscle having greater density (*p* < 0.001). However, at seven days following damage, macrophage density in aged muscle was greater than young (*p* < 0.001). A significant drop in macrophage density between five and seven days in young muscle was observed (*p* = 0.001), while the opposite was observed in aged muscle (*p* < 0.001). The * indicates significant differences detected by a simple main effects post-hoc analysis (*p* < 0.05). *n* = 4–5 per group. Data presented are means ± standard deviation.

Due to the importance of macrophage infiltration during the early stages of the regeneration process, we hypothesized a poor macrophage response, contributing to the poor regeneration observed in aged muscle (Figure 2). Analysis of immunostains using the macrophage-specific marker F4/80 was completed within the necrotic and regenerating regions of muscles (Figure 7A). Surprisingly, in aged TA muscles, macrophage density was found to be greater in the necrotic regions at three and five days following damage (main effect of age: *p* = 0.025; Figure 7B). A significant interaction between age and recovery time was observed in the regenerating regions (*p* < 0.001). At five days following damage, there was a significantly greater macrophage density in young muscle (post-hoc: *p* < 0.001; Figure 7C), while at seven days following damage; macrophage density was greater in aged muscle (post-hoc: *p* < 0.001). Between five and seven days, macrophage density in young muscle declined significantly (post-hoc: *p* < 0.001), whereas in aged muscle, macrophage density increased (Figure 7C).

### 2.5. Expression Pattern and Localization of MMP-9

Although PAI-1 serves as the upstream inhibitor of the plasminogen system, its downstream effectors are proteases which degrade the ECM when activated or allow ECM accumulation if inactivated. In skeletal muscle, the inducible enzyme MMP-9 plays a central role in ECM degradation, thus enabling satellite cell migration and normal regeneration to occur [45,46,47]. In this study, MMP-9 was investigated to better understand the expression pattern and localization of this proteolytic enzyme, and its effects on ECM remodeling.

**Figure 8:**
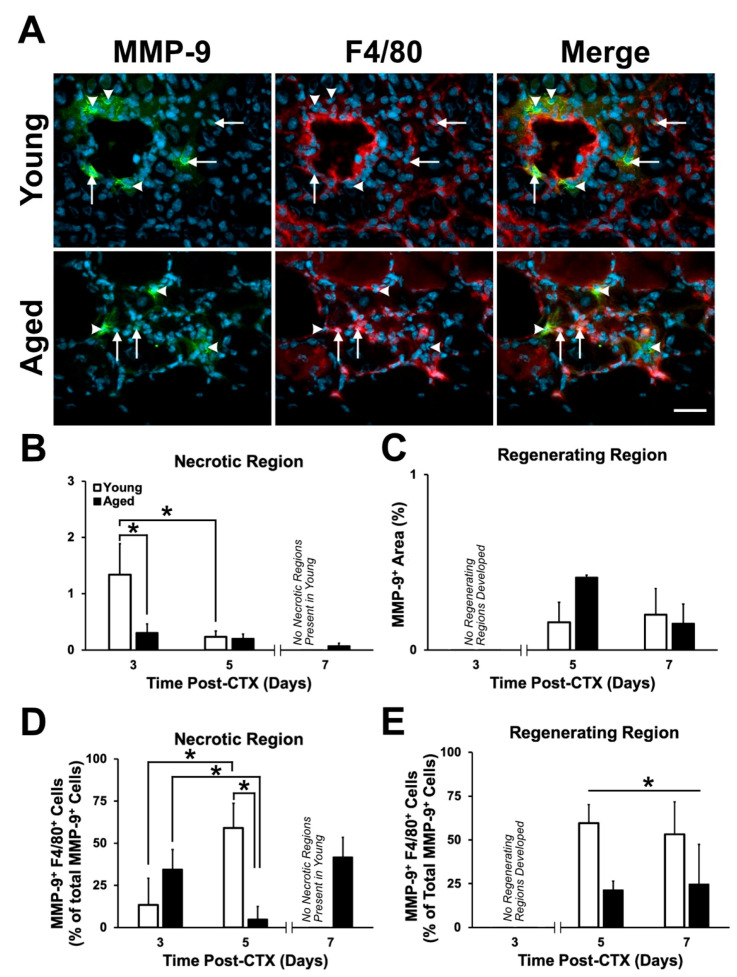
MMP-9 expression was elevated acutely following damage in young muscle. (**A**) Immunostaining of MMP-9 (green), F4/80 (red), and DAPI (blue). Arrows indicate F4/80+/MMP-9+ cells and arrowheads indicate F4/80-/MMP-9+ cells five days post-CTX. (**B**) A significant interaction between age and recovery time point in MMP-9+ area following damage was detected within the necrotic regions (*p* = 0.004). Simple main effect post-hoc analyses demonstrated a significant greater MMP-9 expression within young muscle at three days following damage (*p* < 0.001). MMP-9 expression declined significantly from three to five days following damage (*p* < 0.001). (**C**) No significant differences in MMP-9-positive area were observed in the regenerating regions of young and aged muscle (*p* > 0.05). (**D**) A significant interaction between age and recovery time point on MMP-9+/F4/80+ cells were detected following damage (*p* = 0.004). A simple main effects post-hoc analysis showed significantly greater macrophage-specific (F4/80+) MMP-9 expression in the necrotic regions of young muscle five days following damage (*p* = 0.009). Additionally, macrophage-specific MMP-9 expression increased significantly between three and five days in young, however, the opposite was observed in aged muscle (*p* = 0.026 and *p* = 0.031, respectively). (**E**) A significant main effect of age was found in the regenerating regions, with young muscle displaying a greater percentage of macrophage-specific MMP-9 expression compared to aged muscle (*p* = 0.008). * in (**B**) and (**D**) indicates significance from the simple main effects post-hoc analyses (*p* < 0.05). * in (**E**) indicates a main effect of age. *n* = 4–5 per group. Data presented are means ± standard deviation. Scale bar represents 100 μm.

The proportion of MMP-9-positive area was assessed within the necrotic and regenerating regions of CTX-treated TA muscles (Figure 8A). An interaction effect between age and recovery time on MMP-9 expression was found (*p* = 0.004). MMP-9 was greater three days following damage in the necrotic regions of young compared to aged muscles (post-hoc: *p* < 0.001; Figure 8B). MMP-9 decreased from three to five days following damage in the young muscle only (post-hoc: *p* < 0.001). No significant differences in MMP-9 content was found in the regenerating region (post-hoc: *p* > 0.05; Figure 8C). This is consistent with previous findings suggesting the role of MMPs during the early inflammation stages to enable the degradation of the ECM and facilitate satellite cell migration [45,46,47]. The proportion of MMP-9-positive area was assessed within the necrotic and regenerating regions of CTX-treated TA muscles (Figure 8A). MMP-9 was greater three days following damage in the necrotic regions of young compared to aged muscles (post-hoc: *p* < 0.001; Figure 8B). MMP-9 decreased from three to five days following damage in the young muscle only. No significant differences in MMP-9 content was found in the regenerating regions (Figure 8C). This is consistent with previous findings suggesting the role of MMPs during the early inflammation stages to enable the degradation of the ECM and facilitate satellite cell migration [45,46,47].

Immunostaining of MMP-9 revealed its presence around specific nuclei (Figure 8A). Previously, MMP-9 mRNA was found to be localized primarily in macrophages and polymorphonuclear leukocytes (i.e., neutrophils) in skeletal muscle [45,46,48]. Since aged muscle shows delayed macrophage infiltration and inability to degrade the ECM following damage, it was speculated that aged muscle would display a depression in macrophage-specific MMP-9 expression attributing to the aberrant changes in the ECM. To determine the contribution of macrophages in secreting MMP-9, combination staining of F4/80 and MMP-9 were completed. Total MMP-9+ cells and macrophage (F4/80+) cells colocalized with MMP-9 were counted and analyzed; a significant interaction was found (*p* = 0.004). Post-hoc analysis demonstrated a significant greater percentage of macrophage-specific, MMP-9+ cells five days following damage in the necrotic region of young muscle (post-hoc: *p* = 0.009; Figure 8D). Between three and five days, the proportion of macrophage-specific, MMP-9+ cells decreased significantly in the aged muscle (post-hoc: *p* = 0.031), while the opposite was found in young muscle (post-hoc *p* = 0.026). Young muscle displayed a greater amount of macrophage-specific, MMP-9+ cells in the regenerating regions compared to aged muscle (main effect of age: *p =* 0.008; Figure 8E). Taken together, young muscle acutely displays greater MMP-9 and a greater amount of macrophage-specific, MMP-9+ compared to aged muscle.

### 2.6. Expression Pattern and Localization of PAI-1

Throughout the regeneration process, the modulation of the ECM is needed for the activation, migration, and differentiation of satellite cells [40,42,44]. PAI-1 serves as the upstream inhibitor of the plasminogen system, and has been shown to cause fibrosis, cell senescence, and an impairment in the regeneration process [49,50,51]. Further investigation into the expression pattern and localization of PAI-1 during the acute stages of the regeneration process was undertaken to better understand the role of this regeneration suppressor in aged muscle.

**Figure 9:**
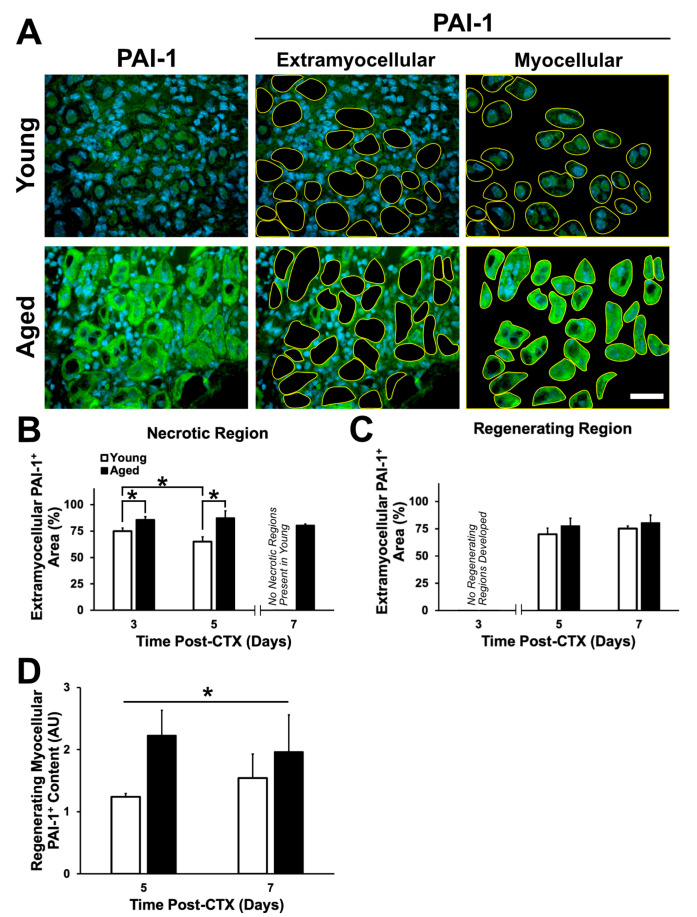
PAI-1 expression is greater in aged skeletal muscle. (**A**) Immunostaining of PAI-1 (green) and DAPI (blue) at five days following damage. (**B**) A significant interaction between age and recovery time point following damage was observed in the necrotic regions (*p* = 0.044). Simple main effect post-hoc analyses demonstrated a significant greater extramyocellular PAI-1 expression within aged muscle at three and five days following damage (*p* = 0.005 and *p* < 0.001, respectively). Extramyocellular PAI-1 within the necrotic regions of young muscle declined significantly between three and five days following damage (*p* = 0.015). * denotes significant differences between groups identified by the simple main effects post-hoc analyses (*p* < 0.05). (**C**) No significant differences in extramyocellular PAI-1 were observed in the regenerating region of young and aged muscle (*p* > 0.05). (**D**) Brightness analysis of PAI-1 within regenerating myofibers showed significantly greater PAI-1 signal within aged regenerating myofibers compared to young (* denotes main effect of age: *p* = 0.029). *n* = 4–5 per group. Data presented are means ± standard deviation. Scale bar represents 50 μm.

Due to the extracellular effect of PAI-1 in delaying the breakdown of the ECM, PAI-1-immunostained TA sections were quantified via area analysis in the distinct necrotic and regenerating regions of the muscle. Extramyocellular PAI-1 was determined through the selection of the area not occupied by myofibers (Figure 9A, middle panels). A significant interaction between age and recovery time was observed (*p* = 0.044). Aged muscle had a significantly greater extramyocellular PAI-1 content at three and five days in the necrotic region compared to young muscle (post-hoc: *p* = 0.005 and *p* < 0.001, respectively; Figure 9B). Interestingly, extramyocellular PAI-1 content decreased significantly between three and five days in young muscle (post-hoc: *p* = 0.015), while in aged muscle, PAI-1 content remained constant. No differences were found in the regenerating regions in aged muscle (Figure 9C). This was consistent with elevated collagen I and macrophage content in the necrotic regions of aged muscle, as previously demonstrated.

During the investigation of extramyocellular PAI-1, regenerating myofibers appeared to be expressing PAI-1. PAI-1 expression within the regenerating myofiber was assessed via analysis of signal intensity, and a significant main effect of age (*p* = 0.029) was found with aged muscle displaying greater PAI-1 within the regenerating myofiber (Figure 9D). This result suggests a role for myocellular PAI-1 in modulating the aberrant changes in the regenerative capacity of skeletal muscle, potentially causing senescence in these myofibers [49].

## 3. Discussion

Aging causes several physiological changes to skeletal muscle leading to functional impairments including reduced muscle strength [52,53]. Accumulating evidence strongly suggests that maintaining a healthy, functional skeletal muscle mass is critical for continued overall health as age progresses [54]; thus, there is considerable interest in developing therapeutic countermeasures against such age-related detriments. Impaired regenerative capacity in skeletal muscle is one avenue that leads to losses in contractile function. However, to date, there are only a limited number of studies investigating the acute responses to damage in aged muscle tissue [10,11,12,13,14,15,16,17,18]. A gap in our knowledge of the effects of age on skeletal muscle regeneration is that no studies have examined an acute time course (i.e., multiple time points) following damage. Therefore, this project was designed to assess aged mouse muscle between three and seven days following CTX-induced muscle damage as these time points encompass several major physiological events—(i) massive turnover of the ECM; (ii) proliferation, migration, and proteolytic function of inflammatory cells; and (iii) proliferation, fusion, and differentiation of myoblasts into de novo myofibers. The findings presented here demonstrate that several aspects of the regeneration process are impaired in aged skeletal muscle in the first week following skeletal muscle damage. Aged muscle lagged behind young skeletal muscle in eliminating necrotic (degenerating) regions of damaged skeletal muscle and in replacing damaged myofibers with de novo eMHC+ myofibres (Figure 2 and Figure 3), consistent with a previous study that noted incomplete regeneration and continued presence of necrotic tissue in aged skeletal muscle following damage [16]. The observation of necrotic and regenerative age-specific changes led us to pursue all further analyses by characterizing necrotic and regenerating regions separately.

### 3.1. Role of PAI-1 in Impaired Muscle Regeneration with Aging

PAI-1 and its control over the plasminogen system potently dictates the success of skeletal muscle regeneration [31,50,55]. Furthermore, PAI-1 is elevated in diabetes (both types 1 and 2) and has been demonstrated to limit tissue regeneration in skeletal muscle [29] and bone [56], which is associated with other diabetic complications such as nephropathy and cardiovascular disease [57,58,59], and induces cellular senescence [49,60,61,62]. Notably, circulating PAI-1 has been demonstrated to increase with advancing age [22,23,24]; however, it is unclear if skeletal muscle itself expresses PAI-1 (whether in an unperturbed state, following exercise, or in response to tissue damage). As well, it is unknown what cell types within skeletal muscle express PAI-1.

For the first time, we have demonstrated local PAI-1 expression in skeletal muscle via immunostaining, in both extracellular and myocellular (i.e., intracellular) spaces (Figure 9). The elevation in extracellular PAI-1 in necrotic aged muscle compared to young muscle was slight but significant. Extracellular PAI-1 affects regeneration by inhibiting plasminogen activators, preventing generation of active plasmin and MMPs, thus limiting proteolytic activity occurring in the extracellular space. This is a critical function during regeneration where a massive number of cells must present and traffic themselves and participate in the degeneration of the ECM and damaged myofibers. In the present study, elevated extramyocellular PAI-1 is consistent with lower MMP-9 levels, aberrant accumulation of collagen I, and the impairment in formation of regenerating myofibers following damage. This is also consistent with the established role of MMP-9 having proteolytic specificity for collagen I [63]. Additionally, PAI-1 has been previously shown to impair macrophage infiltration in damaged muscle [31,64,65,66], and thus, delay the regeneration process. Although aged skeletal muscle had greater macrophage numbers in necrotic regions (Figure 7B), overall, MMP-9 levels were low (Figure 8B), and relatively few MMP-9+ cells were macrophages (Figure 8D). This suggests that the breakdown of necrotic tissue in aged muscle is slow due to, at least in part, impaired proteolytic activity that should be driven by macrophages. In addition to low MMP-9 expression, we hypothesize that elevated PAI-1 is limiting activation of MMP-9 secreted by macrophages in necrotic aged skeletal muscle, preventing progression to regeneration.

Expression of MMP-9 during the early time points of regeneration is critical for the successful degeneration of damaged muscle tissue [45,46,47]. In the current study, MMP-9 expression observed in necrotic and regenerating tissue is predominantly accounted for by macrophages in young but not aged muscle (Figure 8D,E). Other studies have demonstrated MMP-9 expression in damaged skeletal muscle by other cell types, including neutrophils [67,68,69], fibroblasts [70,71,72], and satellite cells [45,73]. Thus, the other MMP-9+ cells can likely be accounted for the combination of these other cell types. 

While not tested in the current study, there are strong data indicating that fibrogenic cell types become more active in aged skeletal muscle [74,75,76,77,78,79]. The accumulation of ECM coupled with prolonged presence of macrophage in the necrotic regions of aged muscle may be a result of either (1) poor clearance of macrophages, or (2) a dysfunctional interplay between macrophages and pro-fibrotic cells. Previous studies have identified fibroadipogenic progenitor cells (FAPs) as major contributors of fat and fibrosis accumulation within skeletal muscle [74,75]. Notably, macrophages within dystrophic muscle was identified to secrete transforming growth factor-β (TGF-β)—termed the master regulator of fibrosis and a known inducer of PAI-1 [80]—within the necrotic regions and in close proximity to FAPs, resulting in differentiation of FAPs into active fibroblasts [76,77,78]. A recent study found that macrophages present within aged human muscle were primarily M2 macrophages [81]. This form of macrophage is able to promote fibrosis through the activation of FAPs [11]. Therefore, it is possible that the accumulation of macrophage that we observed are predominantly M2 macrophages promoting FAP-induced ECM protein expression. Additionally, there may be intrinsic factors within FAPs or systemic factors at play that are outside the functionality of macrophages that might be impairing aged FAP, and therefore, predisposing these cells to a pro-fiberogenic lineage [79]. Further investigation is warranted to better elucidate this mechanism in aged skeletal muscle.

The significance of regenerating myofibers in aged muscle demonstrating a strong intracellular PAI-1 signal is less clear. Given that PAI-1 is largely known for its extracellular roles, it is curious that such an increase in the intracellular (myocellular) space was observed (Figure 9A, D). Several non-mutually exclusive possibilities exist. Intracellular PAI-1 levels could be increased due to: i) greater expression within the myofiber, ii) impaired secretion from the myofiber, iii) increased endocytosis of myofiber- or circulation-sourced PAI-1, and/or iv) impaired degradation of intracellular PAI-1. However, at this point it is unclear which of these possibilities might be occurring. It is noteworthy that PAI-1 transcript levels peak in the first 24 h and remain elevated for 5 days following CTX-induced skeletal muscle damage [82,83] (GEO dataset: GDS234; Reference series: GSE469), consistent with the idea that PAI-1 expression is accelerated. Regardless of the mechanism by which PAI-1 accumulates, the significance of myocellular PAI-1 in aged, regenerating skeletal muscle is unknown. To the best of our knowledge, no studies have examined the potential role for intracellular PAI-1 in skeletal muscle, though has been examined as a negative prognostic factor in ovarian cancer [84] and as a signal to keep hematopoietic stem cells in their niche [80]. 

### 3.2. Alterations to the ECM During Regeneration in Aged Skeletal Muscle

The repercussions of greater intracellular PAI-1 is unclear. Skeletal muscle senescence [60] may be induced by PAI-1 [61,62,85], however, this is likely due to outside-in signaling. Assuming that PAI-1 is not biologically active until secreted, it is likely that greater PAI-1 expression will have its greatest impact on proteolytic activity in the extracellular space. Reduced MMP-9 expression (Figure 8) was hypothesized to lead to accumulation of ECM proteins. Two collagen isoforms (I and IV) were investigated in the present study to provide a better understanding of the changes occurring in the ECM following muscle damage. Previous findings have demonstrated an aberrant accumulation of collagen in aged muscle [35,36,37], however, these studies examined total collagen expression via histochemistry (i.e., no detection of specific isoforms). Results from the present study showed that aged muscle displayed greater collagen I in undamaged and necrotic regions of damaged TA. However, no differences were observed in the regenerating regions between five and seven days following damage. This suggests that collagen I accumulation occurs prior to tissue damage (Figure 4B), is not easily degraded during muscle necrosis (Figure 4C), but is not grossly exacerbated by muscle damage (Figure 4D).

Collagen IV is a major structural component of the basal lamina layer of the basement membrane where it aggregates with other ECM proteins such as laminin to provide structural stability [42,86]. In aged muscle, it is unknown whether collagen IV accumulates and contributes to fibrosis. The current study found no increase in collagen IV expression in aged undamaged or damaged TA using signal threshold analysis. However, the basement membrane was found to be thicker in structure around regenerating myofibers in aged muscle compared to young muscle at the five- and seven-day time points (Figure 5E,F). This is consistent with a recent study on aged human muscle that demonstrated the incarceration of satellite cells by the basement membrane protein laminin [87]. This thick ECM material was shown to impair satellite cell activation, as indicated by fewer MyoD+/Pax7+ cells [87]. Furthermore, our findings show that the collagen IV layer also appeared to have a more irregular shape in aged regenerating skeletal muscle compared to young. The increased thickness and irregular structure (Figure 5E,F) may be due to incomplete breakdown of existing collagen IV and/or accumulation of newly synthesized collagen IV in aged muscle [45], while the absence of an increase in overall collagen IV-positive area may be a result of smaller and less numerous de novo myofibers. The irregular structure of collagen IV may also be a result of improper linking of the basement membrane to the sarcolemma. Thus, we also investigated an important sarcolemmal-ECM linking protein, fibronectin.

Fibronectin binds to collagen IV and other ECM molecules to provide signals to myofibres [88], while satellite cells rely on fibronectin as an adhesion substrate and to drive myogenic signalling during the regeneration process following muscle damage [43,44,89]. The present study found a reduction of fibronectin within necrotic regions of aged muscle during the early phase of the regeneration process (Figure 6C), consistent with our finding of poor myogenic capacity (Figure 1 and Figure 2) and with a recent study that found the genetic ablation of fibronectin in young muscle resulted in a loss of satellite cell number [89]. However, no difference between young and aged muscle fibronectin expression was observed by day 7 of the regeneration process, when the aged muscle had established many de novo myofibers. No differences were found between young and aged muscle in regenerating regions. Taken together, this suggests that poor fibronectin expression contributes to impaired initiation of regeneration.

### 3.3. Study Limitations

Early time points following skeletal muscle damage are critical for initiating successful myogenic events. Currently, no studies have followed up with later time points (e.g., 14, 28 days, or longer) to assess if aged skeletal muscle retains any morphological or functional detriments from injury. The current study examined only early time points, but a follow-up examination of later time points is needed. 

Further, the ECM in skeletal muscle is composed of a diversity of structural (collagen, laminin, elastin) other and smaller molecules of the ground substance (glycosaminoglycans, proteoglycans). However, the current study examined a limited number of ECM components (collagens I and IV, fibronectin). Future work should address other components of the aged skeletal muscle ECM in the post-injury period. 

It would also be of interest to know if there are baseline differences between young and aged control muscle in some of the markers from the current study (e.g., MMP-9, F4/80). Even a slight increase in baseline F4/80+ cells could give rise to far greater numbers upon tissue damage. However, other studies have noted that aging muscle does not have any advantage or disadvantage in resting macrophage number [90,91,92].

Finally, much of this study was observational; comparisons were made between young and aged, necrotic and regenerating skeletal muscle. Future work will need to ascertain that contribution of PAI-1 to the age-related impairments described by our data by way of PAI-1 knockdown or pharmaceutical intervention, as has been successfully implemented in a study of impaired regeneration in type 1 diabetes [29].

## 4. Materials and Methods

### 4.1. Animal Care

Thirty male C57BL/6J mice were acquired from Jackson Laboratory (Bar Harbor, ME, USA) at eight weeks of age. Mice were divided into two groups (*n* = 15 per age group)—young (three months) and aged (18 months) [93]. These groups were then subdivided into three groups to investigate muscle regeneration (*n* = 5 per subgroup)—3, 5, and 7 days post-damage. Male mice were used to avoid the confounding variable of the estrous cycle [94]. The animal room was maintained at 22 °C, 50% humidity, and a 12-h/12-h light-dark cycle. All mice had access to standard mouse chow and water ad libitum. All experimental protocols were performed in accordance with the ethical standards described by the Canadian Council on Animal Care were approved by the University of Windsor Animal Care Committee (AUPP 16-06, approved September 2016).

### 4.2. Skeletal Muscle Damage and Tissue Collection

Skeletal muscle damage was induced with a 50 μL intramuscular injection of 10 μM cardiotoxin (CTX; L8102, Latoxan, France) to the left tibialis anterior (TA) muscles of the mice (at three or 18 months of age). The right TA received no injection and remained undamaged to serve as a control. Mice were euthanized at 3, 5, or 7 days, and the damaged and undamaged TA muscles were carefully dissected away from the lower leg, weighed, and mounted with optimum cutting temperature (OCT) embedding compound on a flat piece of cork. Tissue samples were frozen in isopentane cooled by liquid nitrogen and stored at −80 °C for further analyses.

### 4.3. Histological Analyses

Ten-micron skeletal muscle cross-sections were cut using a Leica CM1860 cryostat (Leica Microsystems Inc., Concord, ON, Canada) and were mounted on glass slides. The subsequent histochemical and immunohistochemical stains are described below.

Hematoxylin and eosin (H&E) stains were used to identify the basic morphology of the muscle following CTX-induced muscle damage. Harris-modified hematoxylin followed by eosin solution (1% *w*/*v* Eosin Y disodium salt in ddH_2_O and 0.1% *v*/*v* glacial acetic acid) were used to stain muscle sections. The resulting stain was used to identify regions of necrotic and regenerating muscle and provided information on the severity of muscle damage during at the specific time points.

Immunostaining was performed to determine the expression pattern (semi-quantitatively) and localization of selected proteins and cells (see Table 1 for antibody list and working concentrations). Muscle sections were fixed using ice-cold 2% paraformaldehyde (PFA) for 5 min at 4 °C and blocked appropriately depending on the protein target. For all rabbit and rat primary antibodies, block consisted of 5% normal goat serum (NGS), and 0.1% Triton-X100 in neutral PBS for one hour at room temperature. When staining for embryonic myosin heavy chain (eMHC), mouse IgG block (BMK2202, Vector Laboratories Inc. Burlingame, CA, USA) was applied according to manufacturer’s instructions for 1 h at room temperature followed by a blocking solution of 10% NGS, 1.5% bovine serum albumin (BSA), and 0.2% Triton-X100 in neutral PBS for one hour at room temperature. Primary antibodies were applied at 4 °C overnight in a humidified chamber. In all staining procedures, a negative control section was also generated (i.e., no primary antibody applied). After washing in PBS, appropriate secondary antibodies were applied along with 4,6-diamidino-2-phenylindole (DAPI) to identify nuclei. A 1:3 dilution of TrueVIEW^®^ (SP-8400, Vector Laboratories Inc., Burlingame, CA, USA) in PBS was used to reduce autofluorescence prior to applying aqueous mounting medium and a cover slip. The eMHC antibody was purchased from the Developmental Studies Hybridoma Bank (DSHB), originally contributed by H.M. Blau.

### 4.4. Image Analysis

H&E stained sections were imaged using a Zeiss Axio Scan Z1 slide scanner equipped with a Hitachi HV F20SCL camera (Carl Zeiss Canada Ltd. Toronto, ON, Canada) Images were taken at a 10× objective (Plan-apochromat 10X/0.45 M27) at a 288% flash intensity and 4 µs flash duration. Subsequent images were then stitched together based on the tissues contrast to the white background and were then Z-stacked and compressed into 1 image using the extended depth of focus. Images obtained were used to assess necrotic and regenerating regions. Regenerating regions were easily identified by the presence of small myofibers with centrally located nuclei. Necrotic regions were identified by the presence of degenerating (i.e., necrotic) myofibers (fragmented borders, lighter H&E stain intensity, and >2 nuclei within the myofiber). Regions that eluded CTX-induced damage were omitted from analysis. Calculation of percentage of damaged area was calculated as follows: 100 × [(necrotic area)/(necrotic + regenerating area)]. Serial cryosections were stained with the described histological and immunostains so that necrotic and regenerating regions could be corroborated between stains/slides.

Images of immunostained sections were obtained using a Nikon 90i eclipse microscope (Nikon Canada, Inc., Mississauga, ON, Canada) and Nikon DS-Qi1Mc camera and analyzed using Nikon Elements Basic Research software (version 3.22, Nikon Canada, Inc., Mississauga ON, Canada). A minimum of five equally distributed images were captured within each of the necrotic and regenerating areas of each muscle. Exposure time was consistent between slides during the imaging process for each immunostain and was determined to be short enough such that the negative control sections displayed no signal.

Analyses were based on determination of average pixel brightness (see Figure 9D), signal thresholding to define positive vs negative regions (see Figure 2, Figure 3, Figure 4, Figure 5, Figure 6 and Figure 9), or manual counting. Collagen and fibronectin analysis used signal thresholding to identify positively stained structures. Collagen IV layer thickness was also assessed. To determine macrophage density, cells were identified using F4/80 cell surface marker and DAPI and were manually counted within the necrotic and regenerating regions. Control (undamaged) TA muscle demonstrated few F4/80+ cells (Figure S2). F4/80+ cells were also assessed for colocalization with MMP-9 via manually counting within their respective regions. Control TA muscle demonstrated virtually no MMP-9 signal (Figure S3). Similarly, PAI-1 was not detectable in control TA muscle. Images were evaluated by two reviewers, independently.

H&E-stained, and eMHC-stained, sections were analyzed for mean myofibre cross-sectional area. Individual fibres were manually outlined to determine cross-sectional area. At least 100 fibers per image, and 3–5 images were analyzed per muscle group.

### 4.5. Statistical Analyses

All data were analyzed using SPSS (IBM SPSS Statistics 25, SPSS Inc., Chicago, IL, USA). A two-way ANOVA was used to analyze for differences in dependent variables between young and aged mice at different time points following induction of muscle damage. The independent variables were age (young or old) and time following muscle damage (3, 5, or 7 days post-damage). If a significant interaction was detected, a post-hoc test consisting of a simple main effects analysis with Bonferroni adjustment was performed to determine the effect of both independent variables on the dependent variable [95]. In some analyses (for example, see Figure 4C,D), insufficient appearance of regenerating regions at three days post-CTX or necrotic regions at seven days post-CTX precluded inclusion of those data in the two-way ANOVA.

A two-tailed *t*-test was used to analyze for potential differences between young and old in the undamaged TA muscle since time post-CTX was irrelevant. All data, including those depicted in figures, are provided as mean ± standard deviation. Outcomes of all statistical analyses (testing for main effects, interactions, post-hoc simple main effects, and *t*-tests) were considered significant at *p* < 0.05. All *p*-values are reported within the results and figure captions. Additionally, a supplementary file is provided with full statistical outputs from SPSS.

## 5. Conclusions

Overall, this study characterized notable alterations to the extracellular environment during skeletal muscle regeneration with aging. Data from this study supports the hypothesis that the regeneration process in aged muscle is impaired as a result of poor remodeling of the ECM. ECM remodeling likely begins immediately following damage; however, in aged skeletal muscle, PAI-1 is highly expressed and is likely suppressing the proteolytic function of MMP-9, thus slowing the activation of satellite cells, and thus limiting the rate of regeneration. Future studies are needed to better understand the mechanism by which extramyocellular and myocellular PAI-1 regulates muscle regeneration, and in doing so, will aid in the development of therapeutic strategies to help mitigate age-related muscle decline.

## Figures and Tables

**Table 1:** Antibody list used for immunofluorescence. Validation is provided on supplier’s website.

Antibody	Supplier	Catalogue	Host Species	Concentration
Collagen I	Abcam	ab34710	Rabbit	1:200
Collagen IV	Abcam	ab6586	Rabbit	1:300
Fibronectin	Abcam	ab23750	Rabbit	1:300
PAI-1	Abcam	ab66705	Rabbit	1:200
MMP-9	Abcam	ab38898	Rabbit	1:200
eMHC	DSHB	F1.652	Mouse	1:1
F4/80	Abcam	ab90247	Rat	1:100

## Supplementary Materials

Supplementary materials can be found at https://www.mdpi.com/1422-0067/21/13/4575/s1. Figure S1, Damaged TA muscle mass relative to body mass; Figure S2, F4/80 and DAPI staining of young undamaged skeletal muscle; Figure S3, MMP-9 and DAPI staining of young undamaged skeletal muscle.

## Abbreviations

CSA	Cross-sectional Area
CTX	Cardiotoxin
DAPI	4,6-diamidino-2-phenylindole
ECM	Extracellular Matrix
eMHC	Embryonic Myosin Heavy Chain
MMP-9	Matrix Metalloproteinase-9
PAI-1	Plasminogen Activator Inhibitor-1
TA	Tibialis Anterior
TGF-β	Transforming Growth Factor-β
uPA	Urokinase Plasminogen Activator

## References

[1] Heymsfield S.B., Adamek M., Gonzalez M.C., Jia G., Thomas D.M. (2014). Assessing skeletal muscle mass: Historical overview and state of the art. J. Cachexia Sarcopenia Muscle.

[2] White H.K., Petrie C.D., Landschulz W., MacLean D., Taylor A., Lyles K., Wei J.Y., Hoffman A.R., Salvatori R., Ettinger M.P. (2009). Effects of an oral growth hormone secretagogue in older adults. J. Clin. Endocrinol. Metab..

[3] Beasley J.M., Shikany J.M., Thomson C.A. (2013). The role of dietary protein intake in the prevention of sarcopenia of aging. Nutr. Clin. Pract..

[4] Deer R.R., Volpi E. (2015). Protein intake and muscle function in older adults. Curr. Opin. Clin. Nutr. Metab. Care.

[5] Neto W.K., Gama E.F., Rocha L.Y., Ramos C.C., Taets W., Scapini K.B., Ferreira J.B., Rodrigues B., Caperuto É. (2015). Effects of testosterone on lean mass gain in elderly men: Systematic review with meta-analysis of controlled and randomized studies. Age.

[6] Sharples A.P., Hughes D.C., Deane C.S., Saini A., Selman C., Stewart C.E. (2015). Longevity and skeletal muscle mass: The role of IGF signalling, the sirtuins, dietary restriction and protein intake. Aging Cell.

[7] Hawke T.J., Garry D.J. (2001). Myogenic satellite cells: Physiology to molecular biology. J. Appl. Physiol..

[8] Dumont N.A., Bentzinger C.F., Sincennes M.-C., Rudnicki M.A. (2015). Satellite Cells and Skeletal Muscle Regeneration. Compr. Physiol..

[9] Grounds M.D. (1991). Towards Understanding Skeletal Muscle Regeneration. Pathol. Res. Pract..

[10] Mann C.J., Perdiguero E., Kharraz Y., Aguilar S., Pessina P., Serrano A.L., Muñoz-Cánoves P. (2011). Aberrant repair and fibrosis development in skeletal muscle. Skelet. Muscle.

[11] Wang Y., Wehling-Henricks M., Samengo G., Tidball J.G. (2015). Increases of M2a macrophages and fibrosis in aging muscle are influenced by bone marrow aging and negatively regulated by muscle-derived nitric oxide. Aging Cell.

[12] Blau H.M., Cosgrove B.D., Ho A.T.V. (2015). The central role of muscle stem cells in regenerative failure with aging. Nat. Med..

[13] Grounds M.D. (1998). Age-associated changes in the response of skeletal muscle cells to exercise and regeneration. Ann. NY Acad. Sci..

[14] Brack A.S., Rando T.A. (2007). Intrinsic changes and extrinsic influences of myogenic stem cell function during aging. Stem Cell Rev..

[15] Stearns-Reider K.M., D’Amore A., Beezhold K., Rothrauff B., Cavalli L., Wagner W.R., Vorp D.A., Tsamis A., Shinde S., Zhang C. (2017). Aging of the skeletal muscle extracellular matrix drives a stem cell fibrogenic conversion. Aging Cell.

[16] Sadeh M. (1988). Effects of aging on skeletal muscle regeneration. J. Neurol. Sci..

[17] Carlson B.M., Faulkner J.A. (1989). Muscle transplantation between young and old rats: Age of host determines recovery. Am. J. Physiol. Cell Physiol..

[18] Grounds M.D. (1987). Phagocytosis of necrotic muscle in muscle isografts is influenced by the strain, age, and sex of host mice. J. Pathol..

[19] Gürlek A., Bayraktar M., Kirazli S. (2000). Increased plasminogen activator inhibitor-1 activity in offspring of type 2 diabetic patients: Lack of association with plasma insulin levels. Diabetes Care.

[20] Meng X., Nikolic-Paterson D.J., Lan H.Y. (2016). TGF-β: The master regulator of fibrosis. Nat. Rev. Nephrol..

[21] Ma L.-J., Fogo A.B. (2009). PAI-1 and kidney fibrosis. Front. Biosci..

[22] Hashimoto Y., Kobayashi A., Yamazaki N., Sugawara Y., Takada Y., Takada A. (1987). Relationship between age and plasma t-PA, PA-inhibitor, and PA activity. Thromb. Res..

[23] Yamamoto K., Takeshita K., Saito H. (2014). Plasminogen activator inhibitor-1 in aging. Semin. Thromb. Hemost..

[24] Yamamoto K., Takeshita K., Kojima T., Takamatsu J., Saito H. (2005). Aging and plasminogen activator inhibitor-1 (PAI-1) regulation: Implication in the pathogenesis of thrombotic disorders in the elderly. Cardiovasc. Res..

[25] Collen D. (1999). The Plasminogen (Fibrinolytic) System. Thromb. Haemost..

[26] Plow E.F., Herren T., Redlitz A., Miles L.A., Hoover-Plow J.L. (1995). The cell biology of the plasminogen system. FASEB J..

[27] Irigoyen J.P., Muñoz-Cánoves P., Montero L., Koziczak M., Nagamine Y. (1999). The plasminogen activator system: Biology and regulation. Cell. Mol. Life Sci..

[28] Garg K., Boppart M.D. (2016). Influence of exercise and aging on extracellular matrix composition in the skeletal muscle stem cell niche. J. Appl. Physiol..

[29] Krause M.P., Moradi J., Nissar A.A., Riddell M.C., Hawke T.J. (2011). Inhibition of plasminogen activator inhibitor-1 restores skeletal muscle regeneration in untreated type 1 diabetic mice. Diabetes.

[30] Francis R.M., Romeyn C.L., Coughlin A.M., Nagelkirk P.R., Womack C.J., Lemmer J.T. (2014). Age and aerobic training status effects on plasma and skeletal muscle tPA and PAI-1. Eur. J. Appl. Physiol..

[31] Koh T.J., Bryer S.C., Pucci A.M., Sisson T.H. (2005). Mice deficient in plasminogen activator inhibitor-1 have improved skeletal muscle regeneration. Am. J. Physiol. Cell Physiol..

[32] Higazi A.A.-R., Laurent G.J., Shapiro S.D. (2006). Fibrinolysis|Overview. Encyclopedia of Respiratory Medicine.

[33] Krause M.P., Al-Sajee D., D’Souza D.M., Rebalka I.A., Moradi J., Riddell M.C., Hawke T.J. (2013). Impaired Macrophage and Satellite Cell Infiltration Occurs in a Muscle-Specific Fashion Following Injury in Diabetic Skeletal Muscle. PLoS ONE.

[34] Tanaka Y., Kita S., Nishizawa H., Fukuda S., Fujishima Y., Obata Y., Nagao H., Masuda S., Nakamura Y., Shimizu Y. (2019). Adiponectin promotes muscle regeneration through binding to T-cadherin. Sci. Rep..

[35] Goldspink G., Fernandes K., Williams P.E., Wells D.J. (1994). Age-related changes in collagen gene expression in the muscles of mdx dystrophic and normal mice. Neuromuscul. Disord..

[36] Wood L.K., Kayupov E., Gumucio J.P., Mendias C.L., Claflin D.R., Brooks S.V. (2014). Intrinsic stiffness of extracellular matrix increases with age in skeletal muscles of mice. J. Appl. Physiol..

[37] Brack A.S., Conboy M.J., Roy S., Lee M., Kuo C.J., Keller C., Rando T.A. (2007). Increased Wnt signaling during aging alters muscle stem cell fate and increases fibrosis. Science.

[38] Lacraz G., Rouleau A.-J., Couture V., Söllrald T., Drouin G., Veillette N., Grandbois M., Grenier G. (2015). Increased Stiffness in Aged Skeletal Muscle Impairs Muscle Progenitor Cell Proliferative Activity. PLoS ONE.

[39] Kjær M. (2004). Role of Extracellular Matrix in Adaptation of Tendon and Skeletal Muscle to Mechanical Loading. Physiol. Rev..

[40] Thomas K., Engler A.J., Meyer G.A. (2015). Extracellular matrix regulation in the muscle satellite cell niche. Connect. Tissue Res..

[41] Kühl U., Ocalan M., Timpl R., Mayne R., Hay E., von der Mark K. (1984). Role of muscle fibroblasts in the deposition of type-IV collagen in the basal lamina of myotubes. Differentiation.

[42] Gulati A.K., Reddi A.H., Zalewski A.A. (1983). Changes in the basement membrane zone components during skeletal muscle fiber degeneration and regeneration. J. Cell. Biol..

[43] Bentzinger C.F., Wang Y.X., von Maltzahn J., Soleimani V.D., Yin H., Rudnicki M.A. (2013). Fibronectin regulates Wnt7a signaling and satellite cell expansion. Cell Stem Cell.

[44] Calve S., Odelberg S.J., Simon H.-G. (2010). A Transitional Extracellular Matrix Instructs Cell Behavior During Muscle Regeneration. Dev. Biol..

[45] Kherif S., Lafuma C., Dehaupas M., Lachkar S., Fournier J.G., Verdière-Sahuqué M., Fardeau M., Alameddine H.S. (1999). Expression of matrix metalloproteinases 2 and 9 in regenerating skeletal muscle: A study in experimentally injured and mdx muscles. Dev. Biol..

[46] Carmeli E., Moas M., Reznick A.Z., Coleman R. (2004). Matrix metalloproteinases and skeletal muscle: A brief review. Muscle Nerve.

[47] Fukushima K., Nakamura A., Ueda H., Yuasa K., Yoshida K., Takeda S., Ikeda S. (2007). Activation and localization of matrix metalloproteinase-2 and -9 in the skeletal muscle of the muscular dystrophy dog (CXMDJ). BMC Musculoskelet. Disord..

[48] Chen X., Li Y. (2009). Role of matrix metalloproteinases in skeletal muscle. Cell Adhes. Migr..

[49] Eren M., Boe A.E., Klyachko E.A., Vaughan D.E. (2014). Role of plasminogen activator inhibitor-1 in senescence and aging. Semin. Thromb. Hemost..

[50] Naderi J., Bernreuther C., Grabinski N., Putman C.T., Henkel B., Bell G., Glatzel M., Sultan K.R. (2009). Plasminogen Activator Inhibitor Type 1 Up-Regulation Is Associated with Skeletal Muscle Atrophy and Associated Fibrosis. Am. J. Pathol..

[51] Cesari M., Pahor M., Incalzi R.A. (2010). Plasminogen Activator Inhibitor-1 (PAI-1): A Key Factor Linking Fibrinolysis and Age-Related Subclinical and Clinical Conditions. Cardiovasc. Ther..

[52] Ali S., Garcia J.M. (2014). Sarcopenia, cachexia and aging: Diagnosis, mechanisms and therapeutic options-A mini-review. Gerontology.

[53] Muscaritoli M., Anker S.D., Argilés J., Aversa Z., Bauer J.M., Biolo G., Boirie Y., Bosaeus I., Cederholm T., Costelli P. (2010). Consensus definition of sarcopenia, cachexia and pre-cachexia: Joint document elaborated by Special Interest Groups (SIG) “cachexia-anorexia in chronic wasting diseases” and “nutrition in geriatrics”. Clin. Nutr..

[54] Li R., Xia J., Zhang X.I., Gathirua-Mwangi W.G., Guo J., Li Y., McKenzie S., Song Y. (2018). Associations of Muscle Mass and Strength with All-Cause Mortality among US Older Adults. Med. Sci. Sports Exerc..

[55] Sisson T.H., Nguyen M.-H., Yu B., Novak M.L., Simon R.H., Koh T.J. (2009). Urokinase-type plasminogen activator increases hepatocyte growth factor activity required for skeletal muscle regeneration. Blood.

[56] Mao L., Kawao N., Tamura Y., Okumoto K., Okada K., Yano M., Matsuo O., Kaji H. (2014). Plasminogen Activator Inhibitor-1 Is Involved in Impaired Bone Repair Associated with Diabetes in Female Mice. PLoS ONE.

[57] Nicholas S.B., Aguiniga E., Ren Y., Kim J., Wong J., Govindarajan N., Noda M., Wang W., Kawano Y., Collins A. (2005). Plasminogen activator inhibitor-1 deficiency retards diabetic nephropathy. Kidney Int..

[58] Lyon C.J., Hsueh W.A. (2003). Effect of plasminogen activator inhibitor-1 in diabetes mellitus and cardiovascular disease. Am. J. Med..

[59] Goldberg R.B. (2009). Cytokine and Cytokine-Like Inflammation Markers, Endothelial Dysfunction, and Imbalanced Coagulation in Development of Diabetes and Its Complications. J. Clin. Endocrinol. Metab..

[60] Baar M.P., Perdiguero E., Muñoz-Cánoves P., de Keizer P.L. (2018). Musculoskeletal senescence: A moving target ready to be eliminated. Curr. Opin. Pharmacol..

[61] Kortlever R.M., Nijwening J.H., Bernards R. (2008). Transforming Growth Factor-β Requires Its Target Plasminogen Activator Inhibitor-1 for Cytostatic Activity. J. Biol. Chem..

[62] Kortlever R.M., Higgins P.J., Bernards R. (2006). Plasminogen activator inhibitor-1 is a critical downstream target of p53 in the induction of replicative senescence. Nat. Cell Biol..

[63] Bigg H.F., Rowan A.D., Barker M.D., Cawston T.E. (2007). Activity of matrix metalloproteinase-9 against native collagen types I and III. FEBS J..

[64] Novak M.L., Bryer S.C., Cheng M., Nguyen M.-H., Conley K.L., Cunningham A.K., Xue B., Sisson T.H., You J.-S., Hornberger T.A. (2011). Macrophage-specific expression of urokinase-type plasminogen activator promotes skeletal muscle regeneration. J. Immunol..

[65] DiPasquale D.M., Cheng M., Billich W., Huang S.A., van Rooijen N., Hornberger T.A., Koh T.J. (2007). Urokinase-type plasminogen activator and macrophages are required for skeletal muscle hypertrophy in mice. Am. J. Physiol. Cell Physiol..

[66] Fibbi G., Barletta E., Dini G., Del Rosso A., Pucci M., Cerletti M., Del Rosso M. (2001). Cell invasion is affected by differential expression of the urokinase plasminogen activator/urokinase plasminogen activator receptor system in muscle satellite cells from normal and dystrophic patients. Lab. Invest..

[67] Ardi V.C., Kupriyanova T.A., Deryugina E.I., Quigley J.P. (2007). Human neutrophils uniquely release TIMP-free MMP-9 to provide a potent catalytic stimulator of angiogenesis. Proc. Natl. Acad. Sci. USA.

[68] Bradley L.M., Douglass M.F., Chatterjee D., Akira S., Baaten B.J.G. (2012). Matrix Metalloprotease 9 Mediates Neutrophil Migration into the Airways in Response to Influenza Virus-Induced Toll-Like Receptor Signaling. PLoS Pathog..

[69] Chakrabarti S., Zee J.M., Patel K.D. (2006). Regulation of matrix metalloproteinase-9 (MMP-9) in TNF-stimulated neutrophils: Novel pathways for tertiary granule release. J. Leukoc. Biol..

[70] Kobayashi T., Hattori S., Shinkai H. (2003). Matrix metalloproteinases-2 and -9 are secreted from human fibroblasts. Acta Derm. Venereol..

[71] Dayer C., Stamenkovic I. (2015). Recruitment of Matrix Metalloproteinase-9 (MMP-9) to the Fibroblast Cell Surface by Lysyl Hydroxylase 3 (LH3) Triggers Transforming Growth Factor-β (TGF-β) Activation and Fibroblast Differentiation. J. Biol. Chem..

[72] Lindner D., Zietsch C., Becher P.M., Schulze K., Schultheiss H.-P., Tschöpe C., Westermann D. (2012). Differential expression of matrix metalloproteases in human fibroblasts with different origins. Biochem. Res. Int..

[73] Guérin C.W., Holland P.C. (1995). Synthesis and secretion of matrix-degrading metalloproteases by human skeletal muscle satellite cells. Dev. Dyn..

[74] Uezumi A., Fukada S., Yamamoto N., Takeda S., Tsuchida K. (2010). Mesenchymal progenitors distinct from satellite cells contribute to ectopic fat cell formation in skeletal muscle. Nat. Cell Biol..

[75] Uezumi A., Ito T., Morikawa D., Shimizu N., Yoneda T., Segawa M., Yamaguchi M., Ogawa R., Matev M.M., Miyagoe-Suzuki Y. (2011). Fibrosis and adipogenesis originate from a common mesenchymal progenitor in skeletal muscle. J. Cell. Sci..

[76] Contreras O., Cruz-Soca M., Theret M., Soliman H., Tung L.W., Groppa E., Rossi F.M., Brandan E. (2019). Cross-talk between TGF-β and PDGFRα signaling pathways regulates the fate of stromal fibro-adipogenic progenitors. J. Cell. Sci..

[77] Contreras O., Rebolledo D.L., Oyarzún J.E., Olguín H.C., Brandan E. (2016). Connective tissue cells expressing fibro/adipogenic progenitor markers increase under chronic damage: Relevance in fibroblast-myofibroblast differentiation and skeletal muscle fibrosis. Cell Tissue Res..

[78] Lemos D.R., Babaeijandaghi F., Low M., Chang C.-K., Lee S.T., Fiore D., Zhang R.-H., Natarajan A., Nedospasov S.A., Rossi F.M.V. (2015). Nilotinib reduces muscle fibrosis in chronic muscle injury by promoting TNF-mediated apoptosis of fibro/adipogenic progenitors. Nat. Med..

[79] Lukjanenko L., Karaz S., Stuelsatz P., Gurriaran-Rodriguez U., Michaud J., Dammone G., Sizzano F., Mashinchian O., Ancel S., Migliavacca E. (2019). Aging Disrupts Muscle Stem Cell Function by Impairing Matricellular WISP1 Secretion from Fibro-Adipogenic Progenitors. Cell Stem Cell.

[80] Yahata T., Ibrahim A.A., Muguruma Y., Eren M., Shaffer A.M., Watanabe N., Kaneko S., Nakabayashi T., Dan T., Hirayama N. (2017). TGF-β–induced intracellular PAI-1 is responsible for retaining hematopoietic stem cells in the niche. Blood.

[81] Cui C., Driscoll R.K., Piao Y., Chia C.W., Gorospe M., Ferrucci L. (2019). Skewed macrophage polarization in aging skeletal muscle. Aging Cell.

[82] Dadgar S., Wang Z., Johnston H., Kesari A., Nagaraju K., Chen Y.-W., Hill D.A., Partridge T.A., Giri M., Freishtat R.J. (2014). Asynchronous remodeling is a driver of failed regeneration in Duchenne muscular dystrophy. J. Cell Biol..

[83] Zhao P., Iezzi S., Carver E., Dressman D., Gridley T., Sartorelli V., Hoffman E.P. (2002). Slug is a novel downstream target of MyoD. Temporal profiling in muscle regeneration. J. Biol. Chem..

[84] Koensgen D., Stope M.B., Tuerbachova I., Bruennert D., Kohlmann T., Braicu I., Sehouli J., Denkert C., Darb-Esfahani S., Stickeler E. (2018). Expression, Intracellular Localization, and Prognostic Value of Plasminogen Activator Inhibitor 1 and PAI-1 RNA-Binding Protein 1 in Primary and Recurrent Ovarian Cancer: A Study of the Tumor Bank Ovarian Cancer Network. Gynecol. Obstet. Invest..

[85] Eren M., Boe A.E., Murphy S.B., Place A.T., Nagpal V., Morales-Nebreda L., Urich D., Quaggin S.E., Budinger G.R.S., Mutlu G.M. (2014). PAI-1–regulated extracellular proteolysis governs senescence and survival in Klotho mice. Proc. Natl. Acad. Sci. USA.

[86] Pöschl E., Schlötzer-Schrehardt U., Brachvogel B., Saito K., Ninomiya Y., Mayer U. (2004). Collagen IV is essential for basement membrane stability but dispensable for initiation of its assembly during early development. Development.

[87] Nederveen J.P., Joanisse S., Thomas A.C.Q., Snijders T., Manta K., Bell K.E., Phillips S.M., Kumbhare D., Parise G. (2020). Age-related changes to the satellite cell niche are associated with reduced activation following exercise. FASEB J..

[88] Gulati A.K., Reddi A.H., Zalewski A.A. (1982). Distribution of fibronectin in normal and regenerating skeletal muscle. Anat. Rec..

[89] Lukjanenko L., Jung M.J., Hegde N., Perruisseau-Carrier C., Migliavacca E., Rozo M., Karaz S., Jacot G., Schmidt M., Li L. (2016). Loss of fibronectin from the aged stem cell niche affects the regenerative capacity of skeletal muscle in mice. Nat. Med..

[90] Tam C.S., Sparks L.M., Johannsen D.L., Covington J.D., Church T.S., Ravussin E. (2012). Low macrophage accumulation in skeletal muscle of obese type 2 diabetics and elderly subjects. Obesity.

[91] Przybyla B., Gurley C., Harvey J.F., Bearden E., Kortebein P., Evans W.J., Sullivan D.H., Peterson C.A., Dennis R.A. (2006). Aging alters macrophage properties in human skeletal muscle both at rest and in response to acute resistance exercise. Exp. Gerontol..

[92] Villalta S.A., Nguyen H.X., Deng B., Gotoh T., Tidball J.G. (2009). Shifts in macrophage phenotypes and macrophage competition for arginine metabolism affect the severity of muscle pathology in muscular dystrophy. Hum. Mol. Genet..

[93] The Mouse in Biomedical Research.

[94] Zhang X. (2010). Hepatocyte growth factor system in the mouse uterus: Variation across the estrous cycle and regulation by 17-beta-estradiol and progesterone. Biol. Reprod..

[95] Bibby P. (2010). Simple Main Effects. Encyclopedia of Research Design.

