# Supplementary material for: Impaired ECM Remodeling and Macrophage Activity Define Necrosis and Regeneration Following Damage in Aged Skeletal Muscle
Source: Int J Mol Sci. 2020 Jun 27;21(13):4575. doi: 10.3390/ijms21134575 (PMC7369722; doi:10.3390/ijms21134575)
Supplement: Supplementary file 1 [file ijms-21-04575-s001.zip › supplementary 1.pdf]

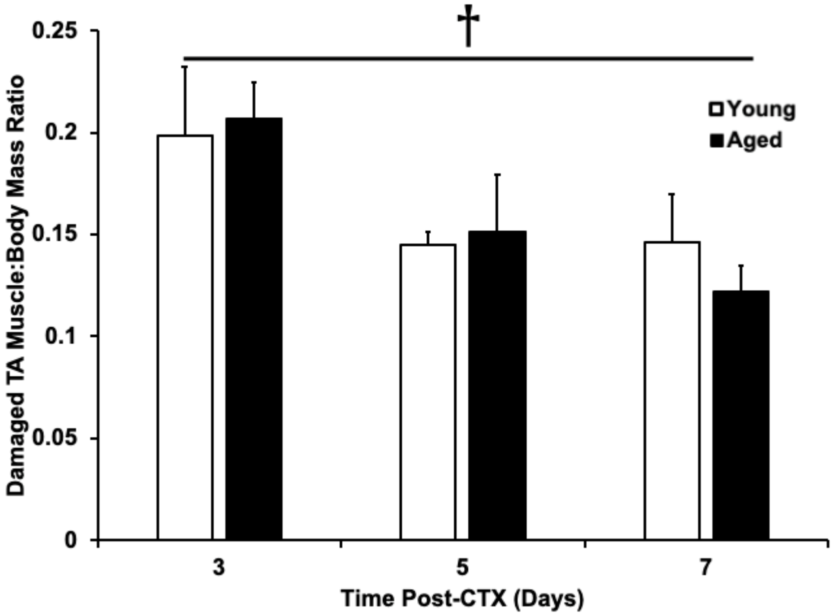

Supplementary Figure 1. Damaged TA muscle mass relative to body mass decreased over time in both groups. No differences in muscle mass to body mass ratio between young and aged groups was observed (p>0.05). † denotes a significant main effect of recovery time point following damage (p<0.001).

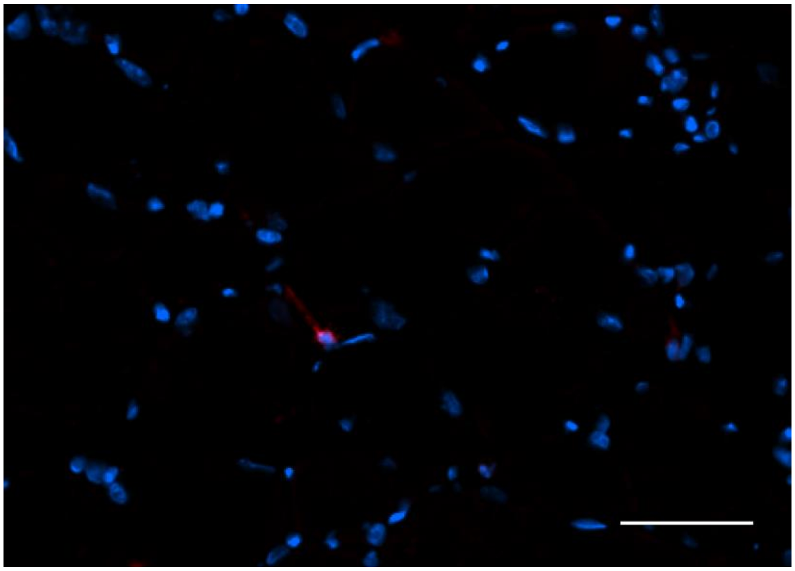

Supplementary Figure 2. F4/80 (red) and DAPI (blue) staining of young undamaged skeletal muscle. Virtually no F4/80 positive cells are present within young undamaged skeletal muscle. Scale bar represents 50μm.

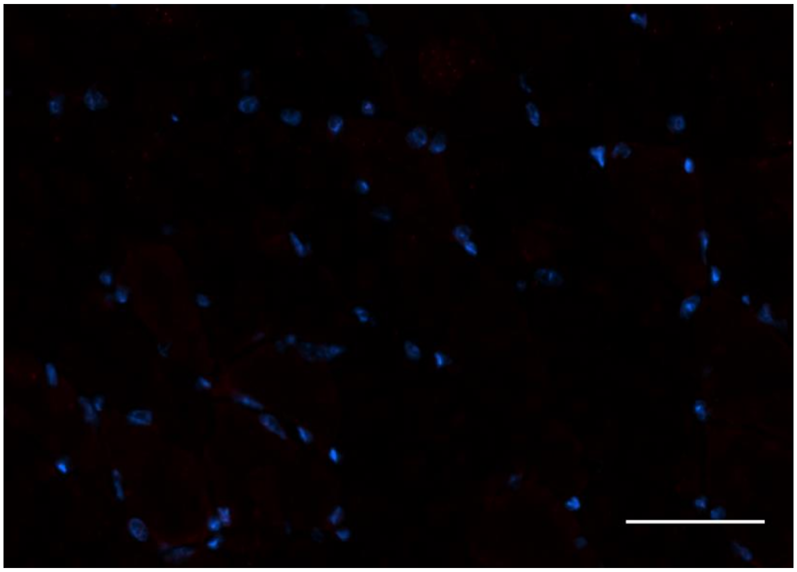

Supplementary Figure 3. MMP-9 (red) and DAPI (blue) staining of young undamaged skeletal muscle. MMP-9 is not present in undamaged skeletal muscle. Scale bar represents 50μm.
